# Will Next-Generation Immunotherapy Overcome the Intrinsic Diversity and Low Immunogenicity of Sarcomas to Improve Clinical Benefit?

**DOI:** 10.3390/cancers12113392

**Published:** 2020-11-16

**Authors:** Hui Yi Chew, Victor Chan, Fiona Simpson, Riccardo Dolcetti

**Affiliations:** 1 Diamantina Institute, Faculty of Medicine, The University of Queensland, Brisbane, QLD 4102, Australia; h.chew@uq.edu.au (H.Y.C.); v.chan1@uqconnect.edu.au (V.C.); f.simpson@uq.edu.au (F.S.); 2 Sir Peter MacCallum Centre for Cancer Immunotherapy, University of Melbourne, Parkville, VIC 3000, Australia

**Keywords:** sarcoma, cancer, immunotherapy, immune checkpoint, cancer vaccine, antibodies, adoptive cell transfer, T cell, natural killer cell

## Abstract

**Simple Summary:**

Treatment modalities for sarcoma have not changed significantly for the past few years despite 25–50% of patients experiencing relapse or progressing to metastatic diseases that become resistant to standard of care therapy, indicating an unmet need for better treatment strategies. While immunotherapy has shown promising results in other types of cancer such as melanoma, first generation immunotherapy trials in sarcomas patients showed unsatisfactory results. Nevertheless, the progressive deepening of our knowledge about the immune landscape of sarcomas and the consequent ability to dissect the heterogeneity of these tumours are leading to a more accurate stratification of patients to be treated with immunotherapy. In addition, new targets are being exploited by a variety of promising immunotherapeutic treatments, which are expected to considerably improve the clinical management of sarcoma patients.

**Abstract:**

Sarcomas are a rare type of a heterogeneous group of tumours arising from mesenchymal cells that form connective tissues. Surgery is the most common treatment for these tumours, but additional neoadjuvant or adjuvant chemotherapy or radiation therapies may be necessary. Unfortunately, a significant proportion of patients treated with conventional therapies will develop metastatic disease that is resistant to therapies. Currently, there is an urgent need to develop more effective and durable therapies for the treatment of sarcomas. In recent years immunotherapies have revolutionised the treatment of a variety of cancers by restoring patient anti-tumour immune responses or through the adoptive infusion of immune effectors able to kill and eliminate malignant cells. The clinicopathologic and genetic heterogeneity of sarcomas, together with the generally low burden of somatic mutations potentially generating neoantigens, are currently limited to broad application of immunotherapy for patients with sarcomas. Nevertheless, a better understanding of the microenvironmental factors hampering the efficacy of immunotherapy and the identification of new and suitable therapeutic targets may help to overcome current limitations. Moreover, the recent advances in the development of immunotherapies based on the direct exploitation or targeting of T cells and/or NK cells may offer new opportunities to improve the treatment of sarcomas, particularly those showing recurrence or resistance to standard of care treatments.

## 1. Introduction

Sarcomas are tumours derived from mesenchymal cells that physiologically give rise to connective tissues, blood vessels, lymphatic vessels, cartilage, and bone. Even though sarcomas are relatively rare as compared to malignancies of epithelial origin, they account for nearly 21% of paediatric solid malignant cancers and less than 1% of adult solid malignancies, and there is little evidence indicating a pathogenic link between environmental or genetic risk factors and incidence [1]. Over 60 different subtypes of sarcoma have been identified based on distinct morphologic and histopathologic features [2]. The two most common types are soft tissue sarcoma (STS) and malignant bone tumours. The most common types of sarcoma affecting adults include leiomyosarcoma, liposarcoma, gastrointestinal stromal tumour (GIST) and fibrosarcoma, whereas Ewing sarcoma, osteosarcoma, synovial sarcoma and rhabdomyosarcoma are commonly seen in children and young adults. The age of the patient can also affect sarcoma severity. For example, fibrosarcoma and Ewing sarcoma are more aggressive in adult patients compared to paediatric patients [3]. Molecular genetic analyses allow further subtyping of these tumours. Generally, sarcomas can be divided into two main groups: (1) a cytogenetically simple group typically characterised by specific, recurrent genomic rearrangements, and (2) a cytogenetically complex group showing complex chromosomal rearrangements, often resulting in copy number alterations [4,5,6]. Sarcomas belonging to the first group include Ewing sarcoma, alveolar rhabdomyosarcoma and synovial sarcoma, whereas the second group includes leiomyosarcoma and undifferentiated pleomorphic sarcoma. Adult sarcomas exhibit increased mutational burden and oncogenic gene fusions are more commonly seen in paediatric sarcomas [7].

The main treatment modalities for sarcoma include surgery, chemotherapy and radiotherapy [8,9,10]. Patients with localised disease undergo surgical resection of the tumour followed by chemotherapy and/or radiotherapy to target residual tumour cells. Adjuvant chemotherapy has markedly improved the five-year survival rate of several types of sarcoma, especially osteosarcoma, Ewing sarcoma and rhabdomyosarcoma, from 20–40% to 60–80% [11]. Despite these multimodality therapies, 25–50% of patients experiences relapse or develop distant metastatic disease that becomes resistant to standard chemotherapy and radiotherapy [12]. The outcome for these patients remains unsatisfactory with no significant improvements observed over previous decades. Therefore, concerted efforts are being made to develop new and more effective therapeutic strategies to control these tumours more effectively. However, the rarity and heterogeneity of this disease make it challenging for the development of novel treatments that may be effective for most subtypes of sarcoma. Furthermore, the relatively low incidence of distinct sarcoma histologies has markedly limited the ability to carry out clinical trials with adequate statistical power. 

The last decade has witnessed an unprecedented revolution in the field of cancer treatment due to the development and clinical application of new immunotherapeutic strategies able to boost or reactivate anti-tumour immune responses. Notably, modulation of immune inhibitory pathways using checkpoint inhibitors has produced durable clinical responses in a sizable subset of patients, leading to their accelerated approval for the treatment of several cancers such as melanoma and renal cell cancer [13]. The clinical success of checkpoint inhibitors has led to a paradigm shift in cancer treatment, clearly indicating that targeting the host’s immune system, rather than tumour, may be more effective than conventional therapies. The development of new and more effective immunotherapies for sarcomas is strictly dependent on a better understanding of the immunogenic features of the different sarcoma subtypes. This is particularly relevant in the light of the marked heterogeneity of sarcomas and the need to develop personalised treatments.

## 2. Immunogenic Landscape of Sarcomas

Immunosurveillance against tumours depends on immune cell recognition of tumour cells through neoantigens generated by somatic mutations or aberrant expression of non-mutated antigens on tumour cells. Under normal conditions, cytotoxic innate and adaptive immune cells such as natural killer (NK) cells and T cells recognise and eliminate tumour cells. However, some tumour cells have evolved the ability to escape immune attack through various mechanisms, such as downregulation of major histocompatibility complex (MHC) from the cell surface and by promoting the formation of an immune suppressive tumour microenvironment (TME). Tumours can recruit immune suppressive immune cells such as myeloid-derived suppressor cells (MDSCs), tumour-associated macrophages (TAMs) and regulatory T cells (Tregs) to promote an immune suppressive TME. The expression of immune checkpoints, either on immune cells or tumour cells, can further dampen the degree of anti-tumour immune response, thus immunotherapy has been widely used to overcome these inhibitory mechanisms and to unleash the immune potential against tumour cells. Current immunotherapeutic strategies include immune checkpoint inhibitors, adoptive T cell transfer, chimeric antigen receptor (CAR) T cells, NK cell-based therapy and therapeutic vaccines. Clinical response to immune checkpoint therapies have been generally associated with tumour microsatellite instability and high tumour mutation burden due to the generation of high numbers of neoantigens, which can be recognised by T cells and mediate elimination of tumour cells. While most sarcoma subtypes have low tumour mutational burden and are widely considered to be non-immunogenic or otherwise known as “immune-cold” tumours, the concept of immunotherapy originally derived from observations made in 1890s by William B. Coley on sarcoma patients [14]. Coley was a bone sarcoma surgeon who injected a mixture of *Streptococcus pyogenes* and *Serratia marcescens*, known as Coley’s Toxin, into his patients with inoperable STS and bone sarcomas to stimulate their immune system, thus attacking the malignant tumours [14]. Imatinib therapy of GIST is another example that further supports the critical involvement of the host’s immune system in mediating therapeutic efficacy. Imatinib is a tyrosine kinase inhibitor that targets KIT and platelet-derived growth factor receptor alpha (PDGFRα) kinases. GIST with *KIT* and *PDGFRA* activating mutations were shown to promote ligand-independent proliferation thereby contributing to the formation of these tumours [15,16,17]. Imatinib was shown to induce 80% objective responses and dramatically improve overall survival (OS) of patients with previously incurable and treatment-resistant GIST [18,19]. While the clinical response of GIST patients treated with imatinib is in part due to inhibition of signalling that drives tumour cell proliferation, a study performed in mouse models reported that imatinib therapy activates CD8^+^ T cells and induces apoptosis of Tregs [20]. This phenomenon was also observed in patient samples where an increase in the ratio of intratumoural CD8^+^ T cells to Treg cells was detected in imatinib-sensitive tumours compared to untreated tumours [20]. This study suggested the potential of combining imatinib therapy with immunotherapy to further enhance the anti-tumour effects. Additionally, Gasparotto et al. examined 82 samples of primary naïve GIST and found that GIST with *KIT* and *PDGFRA* mutations have higher immune infiltration of CD4^+^ and CD8^+^ T cells compared to wildtype GIST [21]. This immune infiltration correlates with higher expression of IFN-γ and components of the antigen presenting machinery, indicating the presence of potential antigen-specific immunity in these tumours. Hedgehog and WNT/β-catenin signalling pathways were predominantly activated in “immune-cold” GIST, suggesting that activation of these immune suppressive signalling pathways hampers infiltration of immune cells into the tumours [21]. Inhibition of Hedgehog and WNT/β-catenin signalling pathways could reverse “immune cold” to “immune hot” GIST [21].

**Figure 1:**
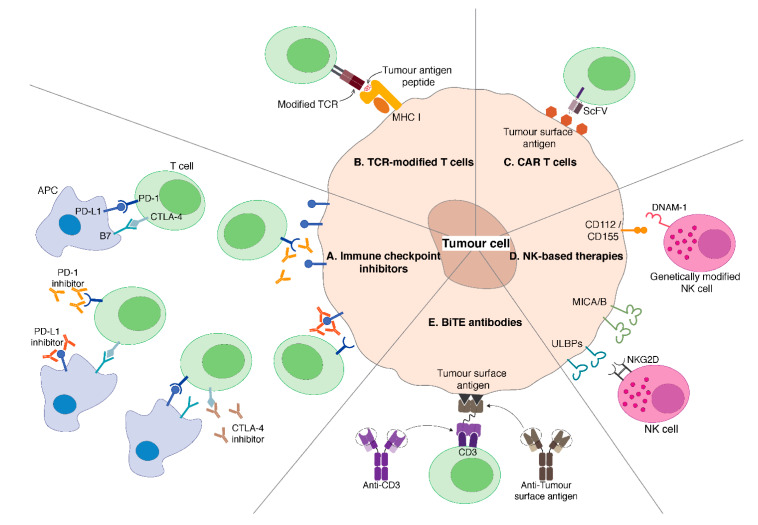
Overview of the different types of T cell and NK cell-based immunotherapies developed for sarcoma treatment. (**A**) The immune checkpoint ligands, PD-L1 and CTLA-4 are expressed on APC and T cells, respectively. Upon engaging with their respective receptors, PD-1 on T cell and B7 on APC, the negative signals dampen the functions of these immune cells thereby preventing the generation of anti-tumour immune responses. PD-L1 can also be overexpressed on tumour cells and prevent T cell-mediated killing. Immune checkpoint inhibitors targeting PD-1, PD-L1 or CTLA-4 can interfere with the engagement between ligands and receptors thereby allowing T cell activation and generation of immune response against tumour cells. (**B**) T cell modified to express TCR against a specific TAA peptide presented on MHC molecules to aid in tumour recognition by the immune cells. (**C**) T cell modified to express CAR, which consists of a monoclonal antibody’s scFv and an intracellular signalling domain, against a specific TAA protein on the tumour cell surface thereby overcoming the issues associated with downregulation of MHC molecules on tumour cells. (**D**) NK cells express activating receptors such as NKG2D and DNAM-1 and they bind to activating ligands, MICA/B, ULBPs and CD112, CD155, respectively, on the tumour cells. NK cells can also be genetically modified to express activating receptors. (**E**) BiTE antibody consists of two domains; one domain recognises TAA on the tumour cell and the second domain recognises CD3 receptor on the T cell, leading to T cell activation. NK, natural killer; PD-L1, programmed cell death-ligand 1; CTLA-4, cytotoxic T-lymphocyte-associated protein 4; PD-1, programmed cell death protein 1; APC, antigen presenting cell; TCR, T cell receptor; TAA, tumour-associated antigen; MHC, major histocompatibility complex; CAR, chimeric antigen receptor; scFv, single-chain variable fragment; BiTE, bispecific T cell engager.

As we continue to uncover the immune landscape of sarcoma and the mechanisms involved in immune tolerance, various cancer immunotherapeutic strategies (Figure 1) can be developed to overcome immune tolerance and immunosuppression thereby improving the current standard of care treatment for sarcoma patients.

## 3. Immune Checkpoint Inhibitors

Evidence accumulated so far indicates that it is important to maintain the balance between positive (activating) and negative (inhibitory) signals when trying to maximise adaptive immune response and still maintaining immunologic tolerance and preventing autoimmunity. These positive and negative signals can be modulated by targeting immune checkpoint molecules. Recently, immune checkpoint blockade studies have shown great potential with extremely durable responses [22]. These agents restore patient’s anti-tumour activity and overcome tumour immune evasion by removing or counteracting the inhibitory signals mediated by distinct immune checkpoint molecules (Figure 1A) [23]. 

### 3.1. CTLA-4 Blockade

CTLA-4 is expressed during the early stage of T cell response, particularly during the primary stage of T cell activation. CTLA-4 also plays an important role in regulating the amplitude of T cell response [24]. CD28 is a costimulatory receptor that is essential in promoting T cell activation and clonal expansion, and CTLA-4 competes with CD28 to bind to B7 ligands on antigen presenting cells (APCs), such as dendritic cells (DCs). CTLA-4/B7 signalling overrides the stimulatory CD28/B7 signalling thus preventing T cell activation, proliferation and proinflammatory cytokines production. CTLA-4 is also expressed on Tregs, which play an important role in modulating immune response and have an immune suppressive impact on adaptive immunity. Blockade of CTLA-4 on Tregs has been shown to abrogate the inhibitory restraints of Tregs on T cells [25]. The potential of inhibiting CTLA-4 for cancer therapy was observed in vivo when administration of anti-CTLA-4 antibodies resulted in rejection of murine colon carcinoma and fibrosarcoma and generation of immunological memory [26]. 

CTLA-4 was the first molecule to be targeted using immune checkpoint inhibitors. Ipilimumab is a clinically available, Food and Drug Administration (FDA)-approved drug against CTLA-4 and has been approved for treatment of advanced melanoma. Preclinical data suggest that paediatric solid tumours express high level of CTLA-4. In a study looking at CTLA-4 expression on a panel of 34 paediatric and adult tumour cell lines, including osteosarcoma and rhabdomyosarcoma, 30 of 34 cell lines showed positive CTLA-4 staining. All of the osteosarcoma and rhabdomyosarcoma cell lines were positive for CTLA-4 expression with osteosarcoma showing higher staining intensity [27]. Furthermore, immunohistochemistry (IHC) was also performed in six human osteosarcoma tissue samples and cytoplasmic and membrane-positive staining was observed in all osteosarcoma samples [27]. Another study looking at peripheral blood samples from 19 paediatric patients (11 osteosarcoma patients and eight Ewing sarcoma patients) reported significantly increased CTLA-4 expression on both CD4^+^ (38% vs. 16%) and CD8^+^ (37% vs. 12%) T cells compared to healthy donors [28].

There has been limited available clinical data regarding the efficacy of ipilimumab in sarcoma. In a phase I study of ipilimumab monotherapy in children, adolescents and young adults with refractory solid malignant tumours (NCT00556881, NCT01445379), 31 patients were available for evaluation. Seventeen were patients with various sarcoma subtypes: eight osteosarcoma, two synovial sarcoma, two clear cell sarcoma, two rhabdomyosarcoma, one pleomorphic sarcoma, one clear cell sarcoma of the kidney, and one undifferentiated sarcoma. Ipilimumab was well tolerated, and stable disease was observed in patients with different sarcoma subtypes, including osteosarcoma and clear cell sarcoma. This study reported increased numbers of activated CD4^+^ T cells with no concomitant expansion of Tregs, and patients with immune-related toxicities showed an increased OS compared to those who did not. Although no objective tumour regression was observed, this study suggests that immune tolerance can be broken in paediatric tumours thus providing a foundation for future combination immunotherapy strategies [29]. A small phase II study assessing ipilimumab in patients with advanced synovial sarcoma was terminated early due to slow patient accrual and lack of clinical efficacy. Among the six patients enrolled in this study, there was no evidence of an immune response and all patients demonstrated disease progression after therapy [30]. The reported median survival was 8.75 months, which was below the average typically observed in patients with metastatic synovial sarcoma [30]. However, due to the rapid progression of disease in these patients, it might have been difficult to observe any potential benefit from ipilimumab treatment, which typically requires time. Another phase II study using a monoclonal antibody against CTLA-4, MDX-010, in patients with advanced synovial sarcoma was also terminated early due to poor accrual (NCT00140855). 

T cells contribute to the anti-tumour effects of imatinib therapy. Synergistic effects were observed in preclinical studies where tumour-bearing mice were treated with the combination of imatinib and anti-CTLA-4 antibody. The combination of imatinib and CTLA-4 blockade significantly reduced tumour size compared to mice treated with monotherapies. A phase Ib study of ipilimumab with dasatinib was performed in 28 patients with GIST and other sarcomas (NCT01643278). This study reported that the combination of dasatinib and ipilimumab is safe; however, limited clinical efficacy was observed for this combination as no partial or complete responses were recorded [31]. 

Single-agent CTLA-4 checkpoint inhibition has not shown very positive results, but immune checkpoint blockade efficacy may depend on the site of action. PD-1 or PD-L1 blockade acts more directly on T cells that are already activated and resides within the TME, which may be more beneficial than single-agent CTLA-4 blockade, which generally enhances T cell priming in tumour draining lymph nodes. 

### 3.2. PD-1/PD-L1 Blockade

PD-1 is another inhibitory receptor expressed during the priming or expansion stage of T cell activation and can also be found on other immune cells such as NK cells and Tregs (Figure 1A). PD-L1, one of the two ligands of PD-1, is expressed on various haematopoietic and non-hematopoietic cell types, including DCs and T cells, and endothelial and epithelial cells, respectively. PD-1/PD-L1 signalling inhibits T cell proliferation, cytokines production and promotes the induction of Tregs. Tumour cells can escape immune destruction by upregulating PD-L1 expression in several tumour types as tumour-infiltrating lymphocytes (TILs) can express high level of PD-1. Blockade of PD-1/PD-L1 axis has demonstrated clinical efficacy in various cancer types, including melanoma. 

Currently, there are three clinically available anti-PD-1 antibodies, namely pembrolizumab, nivolumab and cemiplimab. It was initially hypothesised that the expression of PD-L1 might correlate with worse prognosis and clinical response of PD-1 blockade [32]. PD-L1 expression in sarcoma has been investigated using different antibodies and disparity in results have been reported. 

In a study reported by Kim et al. investigating PD-1 expression on TILs and PD-L1 expression on tumour cells in 105 STS patients, tissue samples reported PD-1 positive TILs and PD-L1-positive tumour cells in 65% and 58% of STS cases, respectively. Expression of PD-1 and PD-L1 was associated with reduced OS and event-free survival [33]. Another study analysed PD-L1 expression in 82 STS patients with various subtypes of STS: rhabdomyosarcoma, synovial sarcoma, Ewing sarcoma, epithelioid sarcoma and mesenchymal chondrosarcoma. Out of 43% of the cases showing positive PD-L1 expression, staining was positive in 100% of epithelioid sarcoma, 53% of synovial sarcoma, 38% of rhabdomyosarcoma and 33% of Ewing sarcoma, whereas PD-L1 was not expressed in any of the mesenchymal chondrosarcoma. This study reported a correlation between PD-L1 expression and shorter OS [34]. Using the VENTANA PD-L1 SP263 kit to investigate the expression of PD-L1 in various types of sarcoma, Vargas et al. reported positive PD-L1 expression (≥1%) in 31% of undifferentiated pleomorphic sarcoma, 29% of angiosarcoma, 26% of rhabdomyosarcoma, 18% of myxofibrosarcoma, 11% of leiomyosarcoma and 10% of dedifferentiated liposarcoma [35]. Negative expression was observed in well-differentiated liposarcoma, myxoid liposarcoma, synovial sarcoma, pleomorphic liposarcoma and Ewing sarcoma. The study also reported higher percentage of PD-L1-positive cells in metastatic/recurrent sarcomas and a significant association between PD-L1 expression and the density of TILs exclusively in leiomyosarcoma but not in other sarcoma subtypes. Several other studies reported contrasting results. D’Angelo et al. used the DAKO 5H-1 antibody to investigate PD-L1 expression on 50 STS patient samples through IHC and reported >1% of PD-L1 expression in 6 of 50 samples [36]. DAKO 5H-1 is also the same antibody used to select patients with PD-1^+^ melanomas and other tumours to be treated with anti-PD-1 antibodies in clinical trials [32,37]. Infiltration of lymphocytes and macrophages was observed in 98% and 90% of the samples, respectively. Twenty-seven of tumour samples showed low density (<5%) of TILs, mainly in patients with leiomyosarcoma, synovial sarcoma and chondrosarcoma, whereas 22 cases had high density (>5%) of TILs, mostly patients with GIST. PD-L1 expression on tumour cells, lymphocytes and macrophages was 12%, 30% and 58%, respectively, with GIST showing the highest prevalence. However, in this study, there was no correlation between PD-L1 expression and clinical outcomes. Based on T cell RNA sequencing, Pollack et al. reported the highest level of T cell infiltration in undifferentiated pleomorphic sarcoma, whereas synovial sarcoma had the lowest, and significantly higher levels of PD-L1 and PD-1 were observed in undifferentiated pleomorphic sarcoma compared to synovial sarcoma [38]. This study also reported no correlation between the expression of PD-1/L1 and progression free survival (PFS) and OS. Issels et al. also reported no association between PD-L1 expression on tumour cells and survival in high-risk localised STS cases [39]. Park et al. examined PD-L1 expression in patient tumour samples with tissue microarray (TMA) analysis carried out with three different antibody clones [40]: ≥1% of PD-L1 expression was observed in 20%, 17.6% and 16.3% of undifferentiated pleomorphic sarcomas with PD-L1 22C3, SP263 and SP142 antibodies, respectively. PD-L1 expression (≥1%) in dedifferentiated liposarcoma was found to be 0% and 3.4% with 22C3 and SP142 antibodies, respectively. Analysis with whole sections showed positive PD-L1 staining in 21.9% of dedifferentiated liposarcoma cases and 3.2% of osteosarcoma cases with PD-L1 22C3 antibody. Significant differences in recurrence-free survival (RFS) and OS rates were observed in dedifferentiated liposarcoma patients as patients with PD-L1 positive tumours had worse RFS and OS compared to those with no PD-L1 expression. However, no significant differences in RFS and OS rates was observed in undifferentiated pleomorphic sarcoma patients with tumours that either expressed or lacked PD-L1. Importantly, this study underlined the issue of the disparity of PD-L1 expression based on the antibodies used in IHC, highlighting the need to standardise the protocol to examine the expression of immune checkpoints before any conclusions about the association with clinical outcomes can be drawn.

SARC028 (NCT02301039) is a completed phase II trial using pembrolizumab monotherapy on advanced sarcoma patients [41]. Seven of the initial 40 STS patient cohort had an objective response, with promising response rates for specific histological subtypes. For 10 patients with undifferentiated pleomorphic sarcoma, one had complete response, three had partial responses. Two of 10 patients with dedifferentiated liposarcoma and 1 of 10 patients with synovial sarcoma showed partial responses. No responses were observed in patients with leiomyosarcoma. This led to cohort expansion in advanced undifferentiated pleomorphic sarcoma and dedifferentiated liposarcoma and an update on long-term outcomes for the two expanded cohorts was presented at 2019 American Society of Clinical Oncology (ASCO) annual meeting. Burgess et al. reported that the undifferentiated pleomorphic sarcoma cohort reached its primary endpoint with an overall response rate (ORR) of 23%, whereas the efficacy of pembrolizumab was not confirmed in the liposarcoma cohort [42]. The lack of response to PD-1 blockade seen in leiomyosarcoma cases was also observed in another phase II study investigating nivolumab monotherapy in uterine leiomyosarcoma cases. No responses were recorded in the 12-patient cohort and the study was terminated early due to lack of efficacy [43]. Correlative analyses were performed on tumour biopsies obtained pre- and during treatment to identify any potential immune features associated with clinical outcomes [44]. Patients who responded to pembrolizumab showed higher numbers of activated T cells and increased PD-L1 positive TAMs in tumours prior to treatment compared to non-responders. Higher numbers of effector memory cytotoxic T cells and Tregs were also observed in tumours at diagnosis compared to non-responders, and the frequencies of both populations increased upon PD-1 blockade. Patients with more Treg cell infiltration at baseline had longer median PFS compared to those with lower Treg cell infiltration. Furthermore, increased density of tumour-infiltrating cytotoxic T cells was associated with better PFS. No clear correlation was observed between expression of PD-L1 and clinical response to pembrolizumab as PD-L1 expression was only positive in 2 of 40 tumour samples. Both PD-L1-positive tumour samples were undifferentiated pleomorphic sarcoma that were responsive to pembrolizumab. However, response was also observed in five patients with other STS subtypes (two undifferentiated pleomorphic sarcoma, two dedifferentiated liposarcoma and one synovial sarcoma) with no PD-L1 tumour expression at baseline. This study showed that PD-L1 is an unreliable predictive marker for PD-1 blockade in STS sarcoma, highlighting the need to identify alternative biomarkers for prediction and patient selection for PD-1 blockade therapy. 

Inconsistent correlations between PD-L1 expression and clinical outcomes were also observed in bone sarcoma patients. Feng et al. investigated TILs and PD-L1 expression in 78 chordomas and reported TILs to be present in 75% of the samples. PD-L1 expression was observed in 94.9% of these tumours. Although the presence of TILs did not correlate with survival, PD-L1 expression significantly correlated with increased numbers of TILs and metastasis [45]. This association was also observed in another study where tumour PD-L1 expression in chordoma was found to correlate with advanced stages and increased TILs content [46]. Machado et al. performed IHC analysis on 370 Ewing sarcoma samples and reported 19.2% of PD-L1^+^ cases, whereas PD-1 expression was observed in 25.7% of samples. Similarly, PD-L1 expression levels were significantly higher in metastatic than primary tumours. TILs were present in 15.4% of the samples. However, there was no correlation between TIL numbers and PD-1/PD-L1 expression and clinical outcome [47]. Raj et al. investigated PD-L1 expression in 240 samples including osteosarcoma, leiomyosarcoma and Ewing sarcoma and reported positive PD-L1 expression in 36%, 97% and 39% of the samples, respectively [48]. This study also reported that PD-L1 expression was associated with positive clinical outcomes [48]. A systematic meta-analysis performed by Zhu et al. reported that PD-L1 expression was associated with OS in osteosarcoma and chondrosarcoma and with event-free survival in both bone sarcoma and STS patients. A correlation between PD-L1 expression and PD-1^+^ TILs was also reported [49]. Another study reported PD-L1 expression in metastatic osteosarcoma but not in the primary tumour. In this series, TILs expressed PD-1 in the metastatic tumours, suggesting that PD-1/PD-L1 axis might be involved in limiting T cell control of metastatic tumours [50]. Koirala et al. reported that PD-L1 expression in osteosarcoma correlated with intra-tumour infiltration of immune cells and event-free survival [51]. This study also reported that PD-L1 positive tumours were more likely to have PD-1 positive immune cell infiltration compared to PD-L1 negative tumours.

Compared to other sarcoma subtypes, osteosarcoma bears higher level of genomic instability and has a greater infiltration of CD8^+^ TILs, which is associated with better prognosis [52,53]. Additionally, PD-L1 expression correlates with poor prognosis suggesting that immune checkpoint inhibitor therapy could be potentially effective in osteosarcoma [51,54,55]. Indeed, preclinical studies of targeting PD-1/PD-L1 axis in osteosarcoma mouse models support the rationale for these therapies. In a humanised mouse model of osteosarcoma, nivolumab treatment induced significantly fewer lung metastasis compared to controls. However, nivolumab did not show any efficacy against primary tumour growth [56]. Lussier et al. also showed antibody-mediated PD-L1 blockade significantly increased survival in tumour-bearing mice and reduced the number of lung metastases compared to control mice [50]. Currently, there are several clinical trials using immune checkpoint inhibitors being conducted in patients with osteosarcoma, such as NCT03006848 and NCT02982486.

Although osteosarcoma was expected to be responsive to immune checkpoint inhibitors, unsatisfactory results were observed in the SARC028 study of pembrolizumab monotherapy administered to advanced sarcoma patients. In the bone sarcoma cohort, objective response was only observed in 2 of 40 patients, including 1 of 22 patients with osteosarcoma and 1 of 5 patients with chondrosarcoma [41]. Recently, Wu et al. investigated the immunogenic potential of osteosarcoma through genomic and IHC analyses, and protein array profiling on 48 osteosarcoma samples, which include primary, relapsed and metastatic tumours [57]. Despite having higher genomic instability than other sarcomas, osteosarcoma does not carry a high load of neoantigens and the mutation burden was not associated with immune infiltration [57]. Comparing to STS subtypes (dedifferentiated liposarcoma and undifferentiated pleomorphic sarcoma) that responded to immune checkpoint inhibitor, osteosarcoma has low immune infiltrates, indicating that the lack of response to immune checkpoint inhibitors might be due to the limited immune cell infiltration [57]. Furthermore, tumours with low immune infiltration had a higher prevalence of deletions, including MHC-encoding genes, whereas adaptive resistance pathways were expressed in tumours with high immune infiltration. This study showed that these immunosuppressive characteristics contribute to the lack of response to PD-1 monotherapy, suggesting that combination therapeutic strategies will be essential to improve clinical outcomes.

### 3.3. CTLA-4 + PD-1/PD-L1 Immune Checkpoint Inhibitors

Several studies explored the potential efficacy of combining different immune checkpoint inhibitors with the goal to increase anti-tumour immunity and clinical response rates. Lussier et al. reported an upregulation of CTLA-4 on tumour-infiltrating T cells in osteosarcoma resistant to anti-PD-L1 antibody therapy [58]. Combination treatment with PD-L1 and CTLA-4 antibodies resulted in complete control of metastatic osteosarcoma in 50% of mice and drastically improved the long-term disease-free survival to 60% compared to 0% observed in mice receiving anti-PD-L1 monotherapy. Combination therapy was also found to increase tumour specific TIL function as compared to monotherapies and induce T cell memory-mediated protection against tumour rechallenge [58]. Alliance A091401 is a multicentre phase II study of nivolumab with or without ipilimumab for metastatic sarcoma. In primary endpoint analysis of the first 76 eligible patients, D’Angelo et al. reported confirmed responses in 2 of 38 patients in nivolumab monotherapy group and 6 of 38 patients in the nivolumab and ipilimumab combination group [59]. The median PFS and OS were 4.1 and 14.3 months for the combination group, respectively, which was an improvement compared to 1.7 and 10.7 months observed in monotherapy group. Responses were observed in angiosarcoma, leiomyosarcoma, myxofibrosarcoma, and undifferentiated pleomorphic sarcoma. The efficacy of nivolumab and ipilimumab combination therapy in bone sarcomas and STS is being investigated in other clinical trials, such as NCT02304458 and NCT02982486.

## 4. Adoptive Transfer of Genetically Modified T Cells

Current immune checkpoint inhibitors work by targeting the inhibitory restraints on the immune system, essentially lifting the “brakes” off and unleashing the anti-tumour immune responses that are already present in the patients. However, in cases in which an active anti-tumour immune response is lacking, simply removing the inhibitory restraints of checkpoint molecules is largely ineffective. Alternative strategies such as adoptive T cell transfer should be considered to provide ex vivo generated anti-tumour immune effectors for these patients. For this strategy, T cells are harvested from the patient and genetically engineered to express transgenic T cell receptor (TCR) that recognise tumour-associated antigen (TAA) peptides presented by MHC molecules (Figure 1B) or chimeric antigen receptor (CAR) that recognise TAA protein expressed on the surface of tumour cells (Figure 1C). Modified T cells are then reinfused back into the patient to mediate tumour destruction. Both types of genetically modified T cells have been explored towards use in the treatment of sarcoma patients. 

### 4.1. T Cells Engineered to Express TAA-Specific TCRs

Cancer testis antigens (CTAs) constitute a group of tumour-associated antigens of high therapeutic relevance due to their low or absent expression in normal tissues and increased expression in neoplastic cells. CTAs are only found in the testes, which are immuno-privileged sites due to the lack of MHC Class I expression. This makes CTAs promising potential targets for immunotherapy as the almost selective expression of CTAs on tumour cells may allow their recognition and elimination by specific T cells [60]. NY-ESO-1 is the most immunogenic CTA and has recently been heavily studied for its potential treatment implications in sarcomas [61]. 

NY-ESO-1 is expressed in 80% of synovial sarcoma [62] and adoptive T cell therapy targeting NY-ESO-1 has been particularly promising in synovial sarcoma compared to other sarcoma subtypes. A clinical trial investigated the efficacy of adoptive T cell transfer with modified TCR targeted against NY-ESO-1 in metastatic melanoma and synovial sarcoma. Objective clinical responses were recorded in 4 of 6 synovial sarcoma patients and 5 of 11 patients with melanoma expressing NY-ESO-1. Complete response was observed in 2 of 11 melanoma patients and one synovial sarcoma patient has a partial response that lasted for 18 months [63]. Based on this promising data, this study then expanded to include 12 additional synovial sarcoma patients and nine melanoma patients. In a follow up study of both patient cohorts, objective clinical responses were observed in 11 of 18 synovial sarcoma patients with 5-year OS rates of 14% and 11 of 20 melanoma patients showed object clinical responses with 5-year OS rates of 33% [64]. A phase I/II study conducted by D’Angelo et al. investigated the safety and efficacy of modified T cells, NY-ESO-1^c259^T cells, that express an affinity-enhanced TCR for NY-ESO-1/LAGE1a-derived peptide, in metastatic synovial sarcoma patients (NCT01343043) [65]. NY-ESO-1 and LAGE-1a are both TAA that share the same peptide presented by human leukocyte antigen HLA-A*02 [66]. From the initial patient cohort, ORR was recorded in 50% of patients. Out of 12 patients, one had confirmed complete response, five had confirmed partial responses, five had stable disease and one had progressive disease. This study also reported that NY-ESO-1^c259^T cells were able to proliferate and produce new effector cells over several months with no sign of exhaustion even after prolonged exposure to antigen [65]. This study expanded to include three additional patient cohorts and in a recent update, 1 of 42 patients had a complete response, 14 of 42 patients had partial response, 24 of 42 patients had stable disease and 3 of 42 patients had progressive disease [67]. This study recruited 10 patients with low antigen expression and observed that 4 of 10 had partial response, five had stable disease and one had progressive disease. Even though there was minimal intra-tumour infiltration of T cells and no detectable expression of PD-L1, high densities of CD163^+^ TAM were observed. Similar to the initial observation [65], modified T cells were able to traffic to tumours and maintained cytotoxic activity 12 months post-infusion. More importantly, this study demonstrated that TAA-specific TCR T cells can be a promising therapeutic strategy to treat non-immunogenic tumours that are resistant to PD-1/PD-L1 blockade [67]. A pilot study examining NY-ESO-1^c259^T cells in advanced myxoid/ round cell liposarcoma is currently underway (NCT02992743).

MAGE-A4 is another CTA expressed by some sarcoma subtypes, including synovial sarcoma and myxoid/round cell liposarcoma. A phase I clinical trial (NCT03132922) investigated the safety and efficacy of T cells expressing an enhanced affinity TCR against MAGE-A4 peptide (ADP-A2M4 SPEAR T cells). In an initial update on eight patients with synovial sarcoma, three had confirmed partial responses, one had unconfirmed partial responses, three had stable disease and one had progressive disease [68]. A recent update on this trial was presented at the 2020 ASCO annual meeting. Hong et al. reported that 7 of 28 patients in the expansion cohort had partial responses, all patients with synovial sarcoma [69]. Data from this study led to the activation of a phase II trial of ADP-A2M4 SPEAR T cells in advanced synovial sarcoma or myxoid/ round cell liposarcoma (NCT04044768).

### 4.2. CAR T Cells

TCR engineered T cells recognise intracellular targets that are presented as immunogenic peptides by MHC molecules; however, many tumours can escape immune recognition by downregulating MHC molecules, thus making them undetectable even by primed T cells. In addition, immunogenic peptides are restricted by their presentation by distinct MHC Class I molecules, thus hampering a broad use of T cells with engineered TCR. To overcome this, T cells can be engineered to express CARs, which recognise surface tumour antigens. First generation CAR T cells carried a chimeric molecule consisting of an antigen recognition ectodomain derived from the single-chain variable fragment (scFv) of a monoclonal antibody and an intracellular signalling domain (Figure 1C). This chimeric receptor gets activated upon binding to the specific antigen expressed at the surface of target cells, thus bypassing the need to be activated through interaction with distinct and patient-specific antigen-MHC complexes. The second and third generation CAR T cells include the addition of one or two co-stimulatory signalling domains, such as CD28, CD137 (4-1BB), and/or CD134 (OX-40) [70], resulting in improved T cell proliferation, survival and anti-tumour immune functions [71]. Initial trials of first generation CAR T cells were disappointing but subsequent generations of CAR T are showing improved efficacy and newer designs are being pursued, each with better functionality and success with reduced immune-related side effects [72].

A phase I clinical trial using first generation anti-disialoganglioside GD2 CAR T cells was tested in patients with refractory neuroblastoma. Despite poor in vivo persistence of CAR T cells, 3 of 11 patients showed complete remission, with two achieving sustained remission [73,74]. Long et al. tested the third generation of anti-GD2 CAR T cells on GD2 positive sarcoma and neuroblastoma cell lines in vitro and reported that GD2 CAR T cells were able to lyse all GD2-positive cell lines effectively [75]. Despite being able to control GD2 positive neuroblastoma effectively in vivo, anti-GD2 CAR T cells showed minimal anti-tumour effect against osteosarcoma tumours in a xenograft mouse model and no improvement in survival was observed. An expansion of myeloid cell populations was observed in sarcoma but not in neuroblastoma xenografts, and this study demonstrated that these myeloid-derived suppressor cells (MDSCs) were responsible for the inhibition of CAR T cell responses in vitro. Treatment with all-trans retinoic acid (ATRA) significantly reduced MDSCs in vivo as ATRA supports the differentiation of immunosuppressive immature myeloid cells into non-suppressive subtype. Combination therapy using ATRA and anti-GD2 CAR T cells significantly enhanced anti-tumour efficacy and improved survival in sarcoma-bearing mice without inducing an increase in Tregs [75]. Chulanetra et al. also examined the efficacy of anti-GD2 CAR T cells against GD2 positive osteosarcoma in vitro and reported anti-GD2 CAR T cells were able to effectively lyse osteosarcoma cells expressing high levels of GD2 [76]. Furthermore, it was reported that the CAR T therapy led to significant increase in the level of PD-L1 expression on tumour cell surface and PD-1 expression of CAR T cells, and that a sub-toxic dose of doxorubicin was able to increase the efficacy of anti-GD2 CAR T cells and reduce tumour PD-L1 expression. These studies demonstrated the potential of CAR T therapy in GD2 positive sarcoma patients and suggested that combination strategies might be the way forward to minimise the effects of immunosuppressive mechanisms induced by tumour cells.

The human epidermal growth factor receptor 2 (HER2) is an additional appealing target for CAR T cell therapy in sarcoma as overexpression of HER2 has been reported in various sarcoma subtypes including osteosarcoma, synovial sarcoma and Ewing sarcoma. Trastuzumab, an anti-HER2 monoclonal antibody, was reported to be ineffective in patients with metastatic osteosarcoma [77]. Despite this, preclinical data obtained in an osteosarcoma model indicate that anti-HER2 CAR T cells may be effective [78]. Ahmed et al. showed that anti-HER2 CAR T cells were able to target HER2-positive osteosarcoma cell lines in vitro and induced regression of primary tumours and metastatic tumours in xenograft mouse model thus prolonging survival [78]. This study demonstrated the potential of anti-HER2 CAR T therapy in tumours that do not express sufficient level of HER2 to be recognised by monoclonal antibodies. A phase I/II clinical trial was conducted using second generation anti-HER2 CAR T cells in patients with HER2-positive recurrent/refractory sarcoma showing that 4 of 17 patients had stable disease without severe toxicity [79]. This study led to another phase I clinical trial (NCT00902044) testing anti-HER2 CAR T cells in combination with lymphodepleting chemotherapy in refractory/metastatic HER2-positive sarcoma patients. In an update of the trial, it was reported that one patient with metastatic rhabdomyosarcoma showed complete response, while stable disease was recorded in 2 of 6 patients and 3 of 6 had progressive disease [80,81]. This warrants further studies in a larger cohort of patients.

Insulin-like growth factor 1 receptor (IGF-1R) and tyrosine kinase orphan-like receptor 1 (ROR1) are highly expressed in various sarcoma cell lines such as alveolar or embryonal rhabdomyosarcoma, Ewing sarcoma, fibrosarcoma and osteosarcoma [82]. Anti-IGF-1R CAR T cells and anti-ROR1 CAR T cells derived from healthy donors showed cytotoxicity against IGF-1R positive and ROR1 positive sarcoma cell lines in vitro, respectively, and were able to release IFN-γ, TNF-α and IL-13 upon antigen stimulation [82]. Anti-IGF1R CAR T cells and anti-ROR1 CAR T cells generated from a sarcoma patient were able to significantly reduce tumour growth in osteosarcoma xenograft mice models [82]. 

Other CAR T cell targets, such as IL-11Rα and B7-H3 have also been tested in various sarcoma subtypes, including osteosarcoma and Ewing sarcoma. Anti-IL-11Rα CAR T cells were effective against primary osteosarcoma and were able to reduce metastatic dissemination with 3 of 5 mice free of pulmonary metastases [83]. The data suggest that anti-IL-11Rα CAR T therapy may be a promising option for osteosarcoma patients with pulmonary metastases. Anti-B7-H3 CAR T cells in vivo studies showed complete regression of established osteosarcoma and Ewing sarcoma tumours in xenograft mice models thus prolonging survival [84]. Anti-B7-H3 CAR T therapy was also able to prolong survival in the metastatic osteosarcoma mice model, suggesting the potential of this therapy in established and metastatic osteosarcoma. 

Studies on CAR T cell therapy for sarcoma demonstrated some promising and exciting data, however, severe adverse effects have been observed, such as cytokine release syndrome, “on-target, off-tumour” toxicity and off-target antigen recognition [85]. Furthermore, CAR T cells might recognise normal cells expressing the target antigens even if they are expressed at very low level. Thus, it is critical to select the target antigen carefully and to monitor early signs of toxicity. 

Studies continue to show compelling evidence on the importance of the immune system in anti-tumour therapeutic strategies to improve patient outcomes. Adoptive T cell therapy has opened up the possibilities of genetically modifying autologous T cells to redirect their activity against molecules that are difficult to target using alternative methods such as monoclonal antibodies. TCR modified T cells and CAR T cells both recognise specific antigens; however, tumours often downregulate their TAA expressions as one of their mechanisms to evade immune recognition, which has been observed in both STS and bone sarcoma [86,87]. One possibility to obviate this limitation is represented by the possible infusion of CAR T cells targeting multiple tumour antigens. Alternatively, NK cell-based immunotherapies can be a valid therapeutic option to overcome the resistance to antigen-specific T-cell responses due to the lack of tumour cell recognition.

## 5. NK Cell-Based Therapies

NK cells are part of the innate immune system and play a crucial role in distinguishing and eliminating infected, stressed and malignant cells. In contrast to T cells, NK cells are able to spontaneously kill tumour cells without any priming or prior activation [88]. 

The number and function of NK cells were mainly investigated in patients with osteosarcoma. While children and adolescent with osteosarcoma have significantly reduced numbers of circulating NK cells at diagnosis [89], adult osteosarcoma patients were shown to retain functional NK cells [90,91]. The recovery of NK cell numbers after standard chemotherapy [92] and the extent of the NK cell expansion induced by IL-2 support during neoadjuvant and adjuvant chemotherapy [93] significantly correlated with enhanced survival of osteosarcoma patients. Moreover, cases expressing low levels of PD-L1 in tumour cells showed enhanced intra-tumour infiltration of NK cells and better survival rates [51]. More recently, the positive impact of NK cells on prognosis was further supported by the enhanced disease-free survival showed by relapsed osteosarcoma patients having a high density of activated NK cells assessed by mRNA and miRNA profiling and CIBERSORT analysis [94].

Activation of NK cells is dependent on the balance between activating and inhibitory signals. One of the main factors mediating the susceptibility of NK cell-mediated lysis is the lack of expression of HLA Class I molecules on target cells, a frequent mechanism of evasion of tumour cells from adaptive immunity, frequently occurring also in sarcoma patients [86]. Indeed, osteosarcoma cell lines with absent or reduced cell surface HLA Class I expression were more efficiently killed by NK cells than tumour cells retaining normal levels of HLA Class I expression [95]. It has been convincingly demonstrated that NK cell activation and killing are also dependent on the strength and duration of the interaction with target cells, effects that are critically mediated by adhesion molecules such as CD54 and CD58 [96,97]. Indeed, downregulation of CD54 was shown to impair the formation of stable interactions between NK cells and osteosarcoma cells, thus favouring immune escape of these tumour cells from NK cell-mediated elimination [98].

The main activating receptors of NK cells include NKG2D, DNAM-1 and natural cytotoxicity receptors (NCRs) such as NKp44 (Figure 1D). Cho et al. investigated NK cell cytotoxicity against various paediatric tumours and observed that Ewing sarcoma and rhabdomyosarcoma cells are sensitive to NK cells in vitro and NK cells were also effective against Ewing sarcoma in vivo thereby prolonging survival. This study demonstrated that NK cell-mediated tumour cell lysis is mediated by NKG2D- and DNAM-1-dependent pathways as treatment of NK cells with anti-NKG2D and anti-DNAM-1 antibodies significantly reduced the cytotoxic activity of NK cells [99]. 

Similar to how tumour cells escape from T cell-mediated lysis, some tumour cells can downregulate NKG2D ligands such as MICA/B and ULBPs, to avoid recognition by NK cells. These findings stimulated the development of strategies able to induce/upregulate the expression of NKG2D ligands on tumour cells to enhance NK cell-mediated tumour cell lysis (Figure 1D). Leung et al. reported that spironolactone (SPIR) is able to upregulate NKG2D ligands in different tumour cell lines, including rhabdomyosarcoma. This resulted in increased NK cell-mediated lysis *in vitro* and suppression of tumour growth in vivo [100]. This study showed that induction of NKG2D ligands on tumour cells is a promising strategy to increase NK cell-mediated lysis on tumour cells. Fernandez et al. also reported moderate to high levels of NKG2D ligands expressed on osteosarcoma cell lines generated from primary and metastatic osteosarcomas. MICA was also expressed at significantly higher levels on metastatic as compared with primary tumours. All of the cell lines tested were sensitive to NK cell-mediated lysis that was shown to be dependent on NKG2D-NKG2D ligand interactions. This study demonstrated that SPIR was able to upregulate NKG2D ligands also in osteosarcoma cells thereby enhancing their sensitivity to NK cell-mediated lysis. Furthermore, activated and expanded NK cells were capable of reducing the number of osteosarcoma-initiating cells in vitro, an effect also dependent on NKG2D-NKG2D ligand interactions. NK cells were also able to reduce tumour burden in vivo and treated mice were free of pulmonary metastases thereby prolonging survival significantly. Combination of NK cells and SPIR treatment further suppressed the tumour growth and increased survival [101]. 

Sayitoglu et al. analysed tumour samples from 32 sarcoma patients and reported that proliferating cell nuclear antigen (PCNA; inhibitory ligand of NKp44) and ligands of DNAM-1, CD112 and CD155, were commonly expressed in these samples [102]. However, characterisation of TILs in freshly dissociated sarcoma samples showed a decrease in the percentage of NK cell population compared to matched PBMCs. Furthermore, the expression of activating receptors, DNAM-1 and NKG2D, were minimal in both peripheral and tumour-infiltrating NK cells. This suggests a general defect in the endogenous NK cell response in sarcoma patients. NK-92 cells were then genetically modified to overexpress one NK cell receptor at a time and subjected to degranulation analysis on 12 selected primary sarcoma explants and two sarcoma cell lines. Gene modified (GM) NK-92 cells expressing DNAM-1 were able to degranulate effectively against all target sarcoma explants and cell lines, GM NK-92 cells expressing NKG2D showed slightly lower degree of degranulation, whereas GM NK-92 cells expressing other activating receptors such as NKp44 and 2B4 showed less than 20% of degranulation. To validate the cytotoxicity of these GM NK-92 cells, these cells were tested against sarcoma explants and it was reported that DNAM-1^+^ GM NK-92 cells were able to significantly increase cytotoxicity compared to wild type NK-92 cells while no significant effect was observed with NKG2D^+^ GM NK-92 cells. This study demonstrated the potential of genetically modifying NK cells to express activating receptors to target sarcoma and other malignancies expressing high levels of activating ligands. 

Currently, there are several clinical trials investigating the safety and efficacy of NK immunotherapy in sarcoma such as NCT02409576 and NCT02849366. 

## 6. Bispecific T Cell Engager (BiTE) Antibodies

Bispecific antibodies are engineered antibodies carrying a TAA recognition domain and a second domain that typically binds to the CD3 molecule expressed at the surface of T cells, thereby promoting their activating and inducing tumour cell killing (Figure 1E). Preclinical data of the BiTE antibody hu3F8-BsAb that targets GD2 in neuroblastomas and melanomas showed promising efficacy data. GD2 was also found to be expressed in STS and osteosarcoma. Xu et al. reported that hu3F8-BsAb was able to suppress tumour progression thus prolonging survival in murine neuroblastoma and melanoma xenograft models [103]. Hu3F8-BsAb was also found to induce T cells and monocytes infiltration into tumour stroma. Currently, hu3F8-BsAb is being tested for safety and efficacy in a phase I/II clinical study in patients with neuroblastoma, osteosarcoma and other solid tumours (NCT03860207). 

## 7. Therapeutic Cancer Vaccines

**Figure 2:**
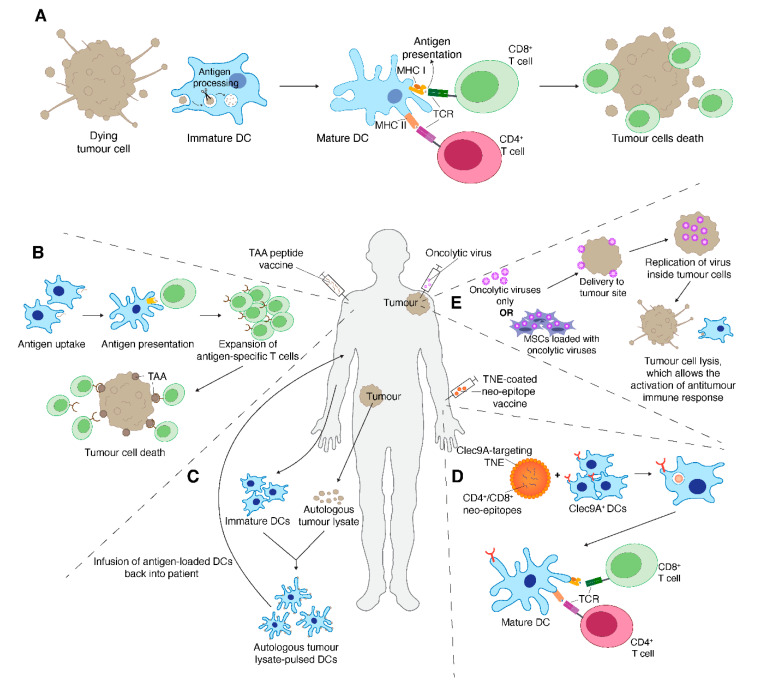
Schematic diagram of therapeutic cancer vaccine and oncolytic virus therapy. (**A**) Immature DCs process captured antigen from a dying tumour cell and undergo maturation. Mature DCs then present antigen to CD8^+^ and CD4^+^ T cell via its MHC I and MHC II molecules, respectively. Activated T cells can now recognise and lyse tumour cells. (**B**) Immunogenic TAA peptides are injected into patients to elicit an antigen-specific immune response. DCs can process and present these peptides to T cells, leading to expansion of antigen-specific T cells which can now target TAA-expressing tumour cells. (**C**) Through surgical resection or biopsy, the tumour sample is collected and processed into tumour lysate. Immature DCs are isolated from patient and pulsed with autologous tumour lysate. These tumour lysate-pulsed mature DCs are then infused back into patient to elicit an anti-tumour immune response. (**D**) CD4^+^ or CD8^+^ neo-epitopes are encapsulated in TNE that are recognised by Clec9A^+^ DCs. Clec9A^+^ DCs then effectively process and present these peptides to CD4^+^ or CD8^+^ T cell via MHC II or MHC I molecules. Activated T cells can now recognise and initiate tumour cell killing. (**E**) Oncolytic viruses are either injected directly into tumours or loaded into mesenchymal stem cells before reinfusion into patients. These viruses undergo replication that eventually causes tumour cell lysis, which allows DCs to capture and uptake tumour antigens to elicit anti-tumour immune response. DC, dendritic cell; MHC, major histocompatibility complex; MSCs, mesenchymal stem cells; TCR, T cell receptor; TAA, tumour-associated antigen; TNE, Tailored NanoEmulsion.

An additional strategy to initiate patient’s anti-tumour immune response is via cancer vaccines (Figure 2). Therapeutic cancer vaccines may exploit different antigenic formulations including genetic material (DNA or RNA), or full-length protein or synthetic (poly)peptides from tumour-associated immunogenic proteins (Figure 2B). [104]. The success of cancer vaccines depends on the selection of immunogenic tumour antigens that will hopefully be able to elicit anti-tumour immune response either through production of tumour-specific antibodies or antigen-specific T cell responses [105,106]. Sarcomas are thought to be ideal for cancer vaccine targets due to the expression of immunogenic antigens such as CTAs, gangliosides and sarcoma-specific fusion proteins generated by chromosomal translocations that are often seen in synovial sarcoma and myxoid/round cell liposarcoma [107].

NY-ESO-1 is the most immunogenic CTA. A HLA-A2 restricted NY-ESO-1 peptide vaccine was found to induce peptide-specific T cell responses and delayed-type hypersensitive (DTH) response in a portion of patients with metastatic NY-ESO-1-positive tumours whom were negative for NY-ESO-1 serum antibodies [108]. The generation of TAA-specific CD8^+^ T cell responses in these patients correlated with disease stabilisation, suggesting the potential of immunisation with NY-ESO-1 peptides [108]. In order to maximise the efficacy of the vaccine and to overcome patients’ HLA type restriction, a study examined a NY-ESO-1 vaccine consisting of the full-length protein formulated with ISCOMATRIX adjuvant (IMX) that elicits strong antibody and T cell responses in patients with resected NY-ESO-1 expressing tumours [109]. This NY-ESO-1 IMX vaccine was able to induce antibody response as well as strong DTH responses, which correlated with survival [109]. CD4^+^ and CD8^+^ T cells that were specific for a wide range of NY-ESO-1 epitopes were also observed in the peripheral, highlighting the potential use of NY-ESO-1 IMX vaccine in various NY-ESO-1 positive tumours [109]. 

A phase II clinical trial examined the use of a trivalent ganglioside vaccine targeting GM2, GD2 and GD3 with OPT821 immunological adjuvant versus OPT821 with placebo in patients with metastatic sarcoma after surgical metastastectomy (NCT01141491). The median PFS was 6.4 months with no significant difference between the treatment groups; however, sustained serologic responses were reported in 98% and 21% of patients in vaccinated and control groups, respectively [110]. 

Chromosomal translocation occurring in distinct subtypes of sarcoma, such as t(X;18)(p11;q11) in synovial sarcoma and t(12;16)(q13;p11) in myxoid/ round cell liposarcoma, can generate tumour-specific immunogenic epitopes that are promising candidates as vaccine targets. The chromosomal translocation in synovial sarcoma results in a SS18-SSX fusion protein [111] and it has been demonstrated that circulating CD8^+^ T cells of HLA-A24^+^ synovial sarcoma patients can recognise the SS18-SSX peptides and mediate tumour-specific immune responses [112,113]. Kawaguchi et al. examined the efficacy of a SS18-SSX peptide fragment vaccine in 21 advanced synovial sarcoma patients [114]. In the peptide fragment alone arm, only 1 of 9 patients did not show disease progression. Another therapy arm included the combination of SS18-SSX peptide fragment with an adjuvant and interferon-alpha (IFN-⍺) and 6 of 12 patients in this therapeutic arm had stable disease. Even though seven patients in this study demonstrated increased numbers of peptide-specific circulating cytotoxic T cells, the treatment did not translate into clinical responses [114]. This indicated that additional immune suppressive mechanisms might be present that prevent the proper function of these cytotoxic T cells. Combination strategies might be necessary to overcome the immune suppressive mechanisms to boost the efficacy of peptide vaccines.

DCs are professional APCs and when primed with tumour antigens, DCs are able to induce anti-tumour immune responses by efficiently presenting the antigens to T cells, which become primed, activated and ready to kill tumour cells (Figure 2A). Thus, one of the main strategies of developing cancer vaccine includes ex vivo generation of autologous DCs, which are loaded with immunogenic peptides and administered back into the patient. Loading autologous DCs with tumour cell lysates or TAA peptides have been tested as therapies in sarcomas (Figure 2C). In a murine model of fibrosarcoma, bone marrow-derived DCs were pulsed with whole tumour lysate and used to immunise mice [115]. This study found that pulsed DCs were able to produce tumour-specific T cell responses and significantly reduced pulmonary metastases, suggesting the potential of tumour lysate-pulsed DCs as therapeutic vaccines in cancer therapy [115]. The ability of tumour lysate-pulsed DCs to stimulate anti-tumour immune response is also supported by another study using LM8 osteosarcoma tumour lysate-pulsed DCs in a murine model [116]. This study reported that administration of LM8-pulsed DCs generated increased antigen-specific cytotoxic T cells activity, enhanced proliferation of CD4^+^ and CD8^+^ T cells and increased serum IFN-γ levels [116].

In a phase I/II clinical study carried out on 37 patients with refractory bone sarcoma and STS, Miwa et al. examined the efficacy of tumour lysate-pulsed DCs immunotherapy [117]. DCs were loaded with autologous tumour lysate and treated with TNF-α and OK-432, which drives maturation of DCs towards activated and functional phenotypes. Significant increase in serum levels of IFN-γ and IL-12 was observed, indicating the activation of immune responses in these patients [117]. Of the 35 evaluable patients, one had partial response, six had stable disease and 28 showed disease progression. The 3-year PFS and OS were 2.9% and 42.3%, respectively. While this study demonstrated that DC-based immunotherapy is safe and able to generate immune responses in sarcoma patients, the results were unsatisfactory and further strategies to enhance DC-based immunotherapy are essential. Another phase I trial used a vaccine composed of autologous tumour lysate-pulsed DCs and DCs treated with keyhole limpet hemocyanin (KLH), an immunogenic carrier protein used as adjuvant, in paediatric solid tumours including osteosarcoma and Ewing sarcoma [118]. This study showed that the vaccine was able to induce antigen-specific T cell responses and one patient with fibrosarcoma exhibited tumour regression in multiple metastatic sites while other sarcoma patients showed disease progression [118]. A phase I clinical trial in patients with relapsed osteosarcoma investigated a vaccine composed of autologous DCs matured with autologous tumour lysate and KLH [119]. Tumour-specific T cell responses were observed in only 2 of 12 patients; however, all three non-osteosarcoma patients showed enhanced specific T cell responses indicating that osteosarcoma patients might be inherently resistant to DC-based immunotherapy [119]. Despite these unsatisfactory clinical results, Merchant et al. treated patients with metastatic and recurrent paediatric sarcomas with autologous tumour lysate/KLH-pulsed DCs with/without recombinant human IL-7, and reported that 62% of patients showed antigen-specific T cell responses [120]. These patients showed prolonged survival with a 5-year OS of 73% compared to 37% of those patients without an immune response, highlighting the potential of this therapeutic strategy to treat high-risk paediatric sarcoma [120]. These clinical studies indicated that administration of autologous tumour lysate-pulsed DCs is a safe therapeutic strategy and is able to activate immune cells in a fraction of cases. Further investigations on how to increase efficacy include combination of DC-based vaccines with a demethylating chemotherapy drug, decitabine, to increase CTAs expression in high-risk sarcomas (NCT01241162) and combination of DC-based vaccines with gemcitabine to inhibit MDSCs (NCT01803152).

In addition to loading autologous DCs with antigens under ex vivo settings, several strategies have been developed to deliver TAAs to DCs in vivo. Dhodapkar et al. reported a strategy to deliver the full-length NY-ESO-1 antigen to DCs in vivo by fusing the TAA to a monoclonal antibody that recognises DEC-205, a receptor expressed by DCs involved in antigen processing and presentation [121]. Phase I trial of this vaccine, CDX-1401, along with Toll-like receptor agonists Resiquimod and Hiltonol to increase antigen-specific T cell responses was administered in patients with advanced refractory tumours, including five patients with sarcoma. Most patients showed serologic response after vaccination, and antigen-specific T cell responses were observed in 56% of patients. While 13 patients showed stable disease and two had tumour regression, none of them carried a sarcoma [121]. However, due to the small population size of sarcoma patients, further investigation in larger patient cohort might be necessary to determine the efficacy of this strategy in sarcoma patients.

Preclinical evidence indicates that one of the major factors limiting the efficacy of cancer vaccines is the indiscriminate presentation of target antigens by mixed populations of DCs, which promote immunogenicity but also tolerance, leading to suboptimal anti-tumour immune responses. This limitation may be overcome by the exploitation of strategies able to deliver tumour antigens to the immunogenic DCs in vivo, an approach that also obviates the complexity and costs that characterise DC-based vaccines. Some of us have developed an innovative Tailored NanoEmulsion (TNE) targeting platform that leverages the superior antigen presenting capacity of Clec9A^+^ “cross-presenting DCs”—a DC population capable of presenting exogenous antigens via MHC II and MHC I, hence stimulating both CD4 and CD8 T-cell responses (Figure 2D) [122]. By exploiting this platform, it has been shown that targeting CD4 and CD8 neo-antigen epitopes to Clec9A^+^ DCs in vivo effectively inhibits the growth of poorly immunogenic tumours [122]. Masterman et al. recently reported another strategy using human CLEC9A antibodies to deliver the NY-ESO-1 antigen to CD141^+^ DCs [123]. This study reported superior response of CLEC9A-NY-ESO-1 to activate antigen-specific CD8^+^ T cells ex vivo in melanoma patients compared to NY-ESO-1 conjugated to DEC-205 antibody or NY-ESO-1 conjugated to control antibody, showcasing the potential of using CLEC9A-NY-ESO-1 antibody to enhance the immune response against NY-ESO-1 positive tumours including sarcomas [123].

Several studies have also investigated the combination of vaccines with immune checkpoint therapies. Kawano et al. investigated the combination of anti-CTLA-4 antibody and DCs pulsed with cryo-treated tumour lysate in a murine osteosarcoma mouse model [124]. This study showed that treatments with anti-CTLA-4 antibody alone and tumour lysate-pulsed DCs alone led to increased intra-tumour infiltration of CD8^+^ T cells, decreased frequency of Treg cells, increased IFN-γ serum levels, reduced burden of pulmonary metastases and prolonged survival [124]. The combination of both treatments was reported to increase the systemic anti-tumour immune response thereby further prolonging the survival of treated mice compared to mice treated with monotherapies [124]. Interim analysis from a phase II trial (NCT02609984) investigating CMB305 vaccine and atezolizumab combination in NY-ESO-1 positive STS demonstrated improved median PFS in the combination arm compared to atezolizumab monotherapy arm (2.6 months and 1.4 months, respectively) [125]. Antigen-specific T cell responses were observed in 53% of patients treated with combination therapy and 25% with atezolizumab monotherapy and 41% of patients in the combination arm had specific antibody responses compared to 0% in the atezolizumab monotherapy arm [125]. An update was presented at the 2019 ASCO annual meeting with no significant differences in PFS and OS between combination versus monotherapy arm, and this study was terminated as it did not reach the efficacy end point [126]. However, patients in the combination arm had more advanced disease and had undergone more chemotherapy treatments than those in the monotherapy group, thus it might be worth investigating the efficacy of combination therapy in patients with less advanced disease stages [126].

## 8. Oncolytic Virus Therapy

In recent years, oncolytic virus therapy has also been investigated as cancer therapy against sarcoma. Oncolytic virus therapy uses viruses that are engineered to selectively replicate in tumour cells. These engineered viruses mediate anti-tumour responses either directly by incorporating viruses into tumour cells which leads to tumour cell lysis or indirectly by promoting anti-tumour immune response as immune cells get exposed to TAA from dying tumour cells (Figure 2E) [127]. In murine preclinical studies, the use of oncolytic virus therapy against sarcoma has showed promising results [128,129,130,131]. Takakuwa et al. utilised herpes simplex virus in murine intraperitoneal fibrosarcoma model and reported 8 of 9 mice remained disease-free and exhibited prolonged survival [131]. These cured mice were also able to reject tumour formation when rechallenged with fresh tumour cells [131]. Another study conducted by Morton et al. tested Seneca Valley virus in solid tumour xenograft models and reported all alveolar rhabdomyosarcoma xenografts showing complete response or maintained complete response [129]. A phase II trial (NCT03069378) examined the efficacy of talimogene laherparepvec (T-VEC), a genetically modified herpes simplex virus in combination with pembrolizumab in patients with advanced or metastatic sarcoma [132]. At the data cutoff date of 24 weeks, the objective response rate (ORR) was 30%; however, a delayed response was observed from a patient at 32-week thus the ORR overall was 35%. Although no complete response was reported, anti-tumour response was observed in distant sites from the sites of T-VEC intratumoural injection, highlighting the potential of this combination therapy for metastatic sarcoma [132]. Studies have also investigated loading oncolytic virus into mesenchymal stem cells (MSCs) to enhance delivery of these viruses to tumour sites and to shield these viruses from inducing anti-viral immune response before reaching the tumour bed [133]. The effects of MSCs have on TME and immune cells still remain largely unknown but Mahasa et al. utilised a mathematical experimental model approach to examine the immune responses elicited by MSCs loaded with oncolytic viruses [134]. This study showed that MSCs loaded with oncolytic virus were able to induce higher tumour killing and decrease tumour burden than treatment with oncolytic virus alone [134]. While limited studies have been performed in sarcoma, it would be interesting to see if this therapeutic approach will be beneficial in sarcoma therapy.

## 9. Future Directions

The field of cancer immunotherapy is constantly evolving and expanding and has demonstrated several exciting breakthroughs over the past decade. Even though such breakthroughs have not been observed in sarcoma thus far, preclinical data and several early clinical trials have reported promising results that warrant further investigation. One of the main challenges observed in immunotherapy is the inability to reliably predict patient response to treatment, thus there is a need for suitable biomarkers to more precisely inform treatment choices. Useful insights to achieve this goal may derive from a better understanding of the complex and heterogeneous TME characterising sarcomas [135,136,137]. A recent immunogenomic study including gene expression data from more than 850 sarcomas had identified three prognosis-related TME groups according to the extent of infiltration of 22 different immune cells [138]. The three TME groups were further investigated for differences in the extent of differentially expressed genes, pathway crosstalk, DNA methylation, copy number variations, and endogenous competitive RNA networks. The TME group with better prognosis was characterised by significantly higher numbers of resting memory CD4^+^ T cells and naïve B lymphocytes. These findings are in keeping with the role of memory CD4^+^ T cells in controlling and sustaining protective immunity and with the function of mature naïve B cells in generating plasma cells secreting antibodies targeting tumour cell antigens. Indeed, recent evidence indicates that the extent of B cell infiltration is the strongest positive prognostic factor in patients with STS, even in cases with low CD8^+^ T cell infiltration [139], suggesting that the immunogenicity of some sarcomas may be driven by B lymphocytes. Taken together, these findings stimulate the development of new strategies able to strengthen the generation of memory CD4^+^ T cells and exploit the anti-tumour functions of B lymphocytes to improve the control of sarcomas. The immunogenomic study also disclosed a strong correlation between functionally active macrophages and a worse prognosis of sarcoma patients [138]. These findings are consistent with an extensive gene expression profiling carried out in 253 STS showing that a M0-macrophage signature correlated with poor metastasis-free survival in all four sarcoma subgroups investigated (synovial sarcoma, myxoid liposarcoma, sarcoma with complex genomic and GIST) [140]. Intriguingly, unlike that observed for other tumours, the infiltration of M2-macrophages characterised either using CD163 gene or the Cibersort M2-Macrophage signature showed no significant prognostic correlation with any of the sarcoma subgroups investigated. These findings point to a pathogenic role of M0-macrophages newly recruited in the TME possibly via CCL2/CCR2 pathway and that may differentiate into M1- or M2-macrophages according to local stimuli. Alternatively, these M0-macrophages may represent a population of uncommitted precursors of resident macrophages. These results provide the rationale to explore new therapeutic strategies for sarcomas based on the targeting of the CSF-1 receptor to promote the differentiation of M0-macrophages into DCs [141].

Another immune cell population with immunosuppressive properties that may be targeted in sarcoma for therapeutic purposes is represented by Tregs whose increased intra-tumour infiltration was shown to correlate with a poor prognosis in cohort of various STS [142]. Recent data demonstrated high expression levels of ICOS and its ligand B7H2 (ICOSL) in GIST, an intercellular interaction promoting the expansion of Tregs that correlated with a poor prognosis in these tumours [140]. Therefore, exploitation of anti-ICOS antagonists, currently under investigation in the clinics (MEDI-570; NCT025250791), could be a promising therapeutic strategy to inhibit Tregs expansion in GIST patients.

Another challenge currently faced is the development of resistance to immunotherapies, which may occur through a variety of different mechanisms both inherent to tumour cells and mediated by cells infiltrating the TME. The lack of anti-PD-1 efficacy in the murine rhabdomyosarcoma model was due to the recruitment of MDSCs to the tumour bed, and disrupting the migration of these MDSCs significantly improved the efficacy of PD-1 blockade [137]. Osteosarcoma tissues were shown to be heavily infiltrated by CXCR4 positive MDSCs which could migrate toward an SDF-1 gradient [143]. The binding of SDF-1 to its cognate CXCR4 receptor induced the downstream activation of the AKT pathway resulting in enhanced survival of MDSCs. Notably, a CXCR4 antagonist was shown to synergise with anti-PD-1 antibody in inhibiting the growth of a murine model of osteosarcoma. These findings strengthen the relevance of new (immuno)therapeutic combinations based on solid preclinical rationale to improve the clinical management of sarcoma.

## 10. Conclusions

Although sarcoma have long been considered “immune cold” tumours, recent efforts have clearly shown a high degree of heterogeneity of the immunogenic features of these tumours. Even if the response rates obtained by first generation clinical trials in sarcoma patients were generally lower as compared to those obtained in other settings, immunotherapy holds great promise to improve the management of patients with sarcomas, as exemplified by the increasing number of ongoing clinical trials (Table 1). Our improved knowledge of the various and complex factors underlying sarcoma immunogenicity will allow a progressive improvement in our ability to stratify patients to optimise the response rates obtainable with immunotherapy. Efforts to better define the mechanisms underlying the inherently poor immunogenicity of some sarcoma subtypes and to identify critical TME immunosuppressive factors may allow the design of new immunotherapeutic strategies able to restore sarcoma immunogenicity. These advances will broaden the spectrum of sarcomas treatable with immunotherapy and pave the way to the design of rational combinations with other forms of treatment able to enhance the overall clinical benefit without hampering host’s immune responses.

## Figures and Tables

**Table 1:** List of ongoing clinical trials for sarcoma immunotherapy.

Trial ID	Phase	Treatment	Sarcoma Type	Status
NCT03074318	I/II	Avelumab and Trabectedin	Advanced liposarcoma and leiomyosarcoma	Active, not recruiting
NCT03006848	II	Avelumab	Recurrent or progressive osteosarcoma	Active, not recruiting
NCT02834013	II	Niovlumab and ipilimumab	Advanced angiosarcoma	Recruiting
NCT03474640	I	Toripalimab	Advanced soft tissue sarcoma and chondrosarcoma	Recruiting
NCT04140526	I	ONC-392 with/without pembrolizumab	Advanced soft tissue sarcoma	Recruiting
NCT02304458	I/II	Nivolumab with/without ipilimumab	Recurrent/refractory sarcoma: Ewing sarcoma, osteosarcoma, rhabdomyosarcoma	Active, not recruiting
NCT04095208	II	Nivolumab with/without relatlimab	Advanced or metastatic soft tissue sarcoma	Recruiting
NCT03899805	II	Eribulin and pembrolizumab	Refractory liposarcoma, leiomyosarcoma, undifferentiated pleomorphic sarcoma	Recruiting
NCT03141684	II	Atezolizumab	Advanced alveolar soft part sarcoma	Recruiting
NCT03338959	I/II	Pembrolizumab with radiation therapy	Intermediate or high-grade soft tissue sarcoma	Recruiting
NCT04458922	II	Atezolizumab	Newly diagnosed/unresectable/metastatic chondrosarcoma, clear cell sarcoma	Recruiting
NCT03307616	II	Neoadjuvant nivolumab, nivolumab and ipilimumab, nivolumab and radiation therapy, nivolumab and ipilimumab and radiation therapy	Recurrent or resectable undifferentiated pleomorphic sarcoma or dedifferentiated liposarcoma	Active, not recruiting
NCT02500797	II	Nivolumab with/without ipilimumab	Metastatic/unresectable bone sarcoma, liposarcoma, undifferentiated pleomorphic sarcoma	Active, not recruiting
NCT03463408	Early I	Neoadjuvant nivolumab and ipilimumab and radiation therapy	Resectable soft tissue sarcoma	Recruiting
NCT03116529	I/II	Neoadjuvant durvalumab and tremelimumab and radiation therapy	High risk soft tissue sarcoma	Recruiting
NCT02815995	II	Durvalumab and tremelimumab	Advanced/metastatic sarcoma	Active, not recruiting
NCT03138161	I/II	Trabectedin and ipilimumab and nivolumab	Advanced/metastatic soft tissue sarcoma	Recruiting
NCT02992743	II	Autologous NY-ESO-1 genetically modified T cells (NY-ESO-1^c259^T)	Advanced myxoid/round cell liposarcoma	Recruiting
NCT04044768	II	Autologous ADP-A2M4 genetically modified T cells	Advanced synovial sarcoma or myxoid/ round cell liposarcoma	Recruiting
NCT03450122	I	Autologous NY-ESO-1 genetically modified T cells and chemotherapy and aldesleukin with/without immunostimulatory agents: CMB305 and/or antigen-specific vaccine (ID-LV305)	Advanced or recurrent synovial sarcoma, myxoid liposarcoma, NY-ESO-1 positive sarcoma	Recruiting
NCT02650986	I/II	Autologous NY-ESO-1 genetically modified T cells with/without decitabine	Advanced/metastatic/unresectable synovial sarcoma	Recruiting
NCT03250325	I/II	Autologous NY-ESO-1 genetically modified T cells	Unresectable NY-ESO-1 positive synovial sarcoma	Active, not recruiting
NCT04556669	I	Autologous CD22 CAR genetically modified T cells or TILs with scFv fragment of anti-PD-L1 monoclonal antibody	Sarcoma	Recruiting
NCT03635632	I	Autologous GD2 CAR genetically modified T cells	Relapsed GD2 positive osteosarcoma, Ewing sarcoma, rhabdomyosarcoma	Recruiting
NCT03635632	I/II	Autologous sarcoma specific (CD133, GD2, Muc1, CD117 or other marker) CAR genetically modified T cells	Advanced/ recurrent sarcoma	Recruiting
NCT03638206	I/II	Autologous NY-ESO-1 CAR genetically modified T cells	Synovial sarcoma	Recruiting
NCT00902044	I	Autologous HER2-CD28 CAR genetically modified T cells	Refractory HER2 positive sarcoma, metastatic HER2 positive osteosarcoma	Active, not recruiting
NCT04483778	I	Autologous B7H3 CAR or bispecific B7H3 and CD19 CAR genetically modified T cells	Osteosarcoma, Ewing sarcoma, rhabdomyosarcoma, synovial sarcoma, clear cell sarcoma, soft tissue sarcoma	Recruiting
NCT04433221	I/II	Autologous sarcoma specific (GD2, HER2, PSMA, CD276 or other marker) CAR genetically modified T cells	Advanced/recurrent sarcoma	Recruiting
NCT02100891	II	HLA-haploidentical haematopoietic cell transplantation and donor NK cell infusion	Advanced/recurrent Ewing sarcoma, rhabdomyosarcoma, osteosarcoma	Active, not recruiting
NCT02409576	I/II	Expanded and activated allogenic NK cells	Advanced/metastatic/relapsed Ewing sarcoma, rhabdomyosarcoma	Recruiting
NCT03860207	I/II	Humanised 3F8 bispecific antibody (Hu3F8-BsAb)	Relapsed/refractory GD2 positive osteosarcoma	Recruiting
NCT01803152	I	Autologous tumour lysate with dendritic cell vaccine with/without myeloid derived suppressor cells inhibition	Relapsed sarcoma	Active, not recruiting
